# Programmed Cell Death and Autophagy in an *in vitro* Model of Spontaneous Neuroretinal Degeneration

**DOI:** 10.3389/fnana.2022.812487

**Published:** 2022-02-11

**Authors:** Kevin Puertas-Neyra, Nadia Galindo-Cabello, Leticia A. Hernández-Rodríguez, Fernando González-Pérez, José Carlos Rodríguez-Cabello, Rogelio González-Sarmiento, José Carlos Pastor, Ricardo Usategui-Martín, Ivan Fernandez-Bueno

**Affiliations:** ^1^Retina Group, Instituto Universitario de Oftalmobiología Aplicada (IOBA), Universidad de Valladolid, Valladolid, Spain; ^2^Postgraduate Unit, Faculty of Biological Sciences, National University of San Marcos, Lima, Peru; ^3^Group for Advanced Materials and Nanobiotechnology (GIR BIOFORGE), CIBER-BBN, Edificio LUCIA, Universidad de Valladolid, Valladolid, Spain; ^4^Centro en Red de Medicina Regenerativa y Terapia Celular de Castilla y León, Valladolid, Spain; ^5^Molecular Medicine Unit, Department of Medicine, University of Salamanca, Salamanca, Spain; ^6^Institute of Biomedical Research of Salamanca (IBSAL), Salamanca, Spain; ^7^Institute of Molecular and Cellular Biology of Cancer (IBMCC), University of Salamanca-CSIC, Salamanca, Spain; ^8^Red Temática de Investigación Cooperativa en Salud (RETICS), Oftared, Instituto de Salud Carlos III, Valladolid, Spain; ^9^RetiBrain (RED2018-102499-T), Ministerio de Ciencia, Innovación y Universidades, Valladolid, Spain

**Keywords:** retina, neurodegeneration, autophagy, apoptosis, necroptosis

## Abstract

Retinal neurodegenerative diseases are the leading causes of visual impairment and irreversible blindness worldwide. Although the retinal response to injury remains closely similar between different retinal neurodegenerative diseases, available therapeutic alternatives are only palliative, too expensive, or very specific, such as gene therapy. In that sense, the development of broad-spectrum neuroprotective therapies seems to be an excellent option. In this regard, it is essential to identify molecular targets involved in retinal degeneration, such as cell death mechanisms. Apoptosis has been considered as the primary cell death mechanism during retinal degeneration; however, recent studies have demonstrated that the only use of anti-apoptotic drugs is not enough to confer good neuroprotection in terms of cell viability and preservation. For that reason, the interrelationship that exists between apoptosis and other cell death mechanisms needs to be characterized deeply to design future therapeutic options that simultaneously block the main cell death pathways. In that sense, the study aimed to characterize the programmed cell death (in terms of apoptosis and necroptosis) and autophagy response and modulation in retinal neurodegenerative diseases, using an *in vitro* model of spontaneous retinal neurodegeneration. For that purpose, we measured the mRNA relative expression through qPCR of a selected pool of genes involved in apoptosis (*BAX*, *BCL2*, *CASP3*, *CASP8*, and *CASP9*), necroptosis (*MLKL*, *RIPK1*, and *RIPK3*), and autophagy (*ATG7*, *BCLIN1*, *LC3B*, *mTOR*, and *SQSTM1*); besides, the immunoexpression of their encoding proteins (Casp3, MLKL, RIPK1, LC3B, and p62) were analyzed using immunohistochemistry. Our results showed an increase of pro-apoptotic and pro-necroptotic related genes and proteins during *in vitro* retinal neurodegeneration. Besides, we describe for the first time the modulation between programmed cell death mechanisms and autophagy in an *in vitro* retinal neurodegeneration model. This study reinforces the idea that cell death mechanisms are closely interconnected and provides new information about molecular signaling and autophagy along the retinal degeneration process.

## Introduction

Retinal neurodegenerative diseases (RND) are the leading causes of irreversible blindness worldwide. The most representative RND is Age-related Macular Degeneration (AMD), which affects up to 8.7% of the global population over 65 years-old being the third cause of visual impairment globally (Wong et al., 2014; Bourne et al., 2021; Steinmetz et al., 2021). RND causes a severe and irreversible impact on the quality of life of the patients and an enormous financial global burden (Bourne et al., 2021; Steinmetz et al., 2021). At present, there is no curative treatment available for RND apart from costly gene therapy for very selective mutations (Russell et al., 2017; Cehajic-Kapetanovic et al., 2020). However, despite the etiology, retinal response to injury in terms of cellular signalization pathways remains closely similar for most RND (Cuenca et al., 2014). In that sense, looking for common therapeutic molecular targets through the characterization of the different cell death mechanisms could be an excellent therapeutic option for many of these diseases.

Retinal response to injury includes promoting inflammatory response, activation of the antioxidant machinery, and programmed cell death mechanisms (Cuenca et al., 2014). Cell death events during retinal degeneration have been associated predominantly with the activation of apoptosis as the primary programmed cell death mechanism (Barber et al., 1998; Choudhury et al., 2013; Comitato et al., 2014; Radi et al., 2014). However, recent studies have highlighted the importance of other programmed cell death mechanisms, such as necroptosis. Besides, the modulation of autophagy, an essential physiological catabolic mechanism, has also been associated with retinal neurodegeneration (Murakami et al., 2013; Newton and Megaw, 2020). The importance and predominance of these mechanisms vary according to the RND. For instance, novel studies have highlighted the importance of necroptosis during the pathophysiology of the Retinitis Pigmentosa (RP) (Arango-Gonzalez et al., 2014; Viringipurampeer et al., 2019) and autophagy in AMD (Mitter et al., 2012; Kaarniranta et al., 2017). The importance of non-apoptotic cell death mechanisms and autophagy have been confirmed *in vivo*, in which molecular targeting studies have failed to prevent cell death by blocking only the apoptotic pathways (Hisatomi et al., 2001; Zacks et al., 2003; Trichonas et al., 2010). This background suggests a complex interrelationship between these mechanisms during retinal degeneration. However, the implications of these interactions remain unclear (Newton and Megaw, 2020).

*In vitro* retinal explant cultures are helpful tools for studying neuroretinal degeneration processes because they replicate, with *in vitro* limitations, the cellular and molecular changes that occur in retina degeneration (Li et al., 2018; Murali et al., 2019; Fernandez-Bueno and Usategui-Martin, 2021). Although this model does not mimic a specific retinal disease, it resembles neurodegeneration, a joint event in most RND (Cuenca et al., 2014).

Describing the programmed cell death mechanisms and other cell processes involved in retinal neurodegeneration is essential to identify critical molecular targets to develop unspecific neuroprotective therapies for RND. In this scenario, this study aimed to characterize the programmed cell death and autophagy mechanisms in neuroretina using an *in vitro* spontaneous neuroretinal degeneration model.

## Materials and Methods

### *In vitro* Model of Spontaneous Neuroretinal Degeneration

Organ neuroretinal explants cultures were used as a spontaneous neuroretinal degeneration model described by Fernandez-Bueno and Usategui-Martin (2021). As a summary, we used fresh eyes extracted from pigs 6 to 8 months derived from the local slaughterhouse. Two neuroretinal explants (5 × 5 mm) derived from the *area centralis* (cone-enriched visual streak without blood vessels) were extracted from each eye (Supplementary Figure 1) and placed over the porous membrane of a Transwell plate (24 mm diameter, 0.4 mm pore; Corning Life Sciences, Corning, NY, United States) with the photoreceptor layer facing the membrane.

Cultures were maintained in 1.5 ml of a medium composed by Neurobasal A supplemented with 10% fetal bovine serum (FBS), 1% antibiotic-antifungal mixture, 2% B-27, and 1% L-glutamine (Gibco, Invitrogen, Paisley, United Kingdom) under standard conditions (37°C, 5% CO_2_ atmosphere) for 1, 3, 6, and 9 days. In parallel, neuroretinal explants were extracted and processed directly as negative control (fresh neuroretina). Experimental conditions were determined as fresh neuroretina, 1, 3, 6, and 9 days of neuroretinal degeneration. We ran experiments in triplicate; three retinal explants were used for each experimental condition, and thus, we used a total number of 15 retinal explants.

### RNA Extraction, Reverse Transcription, and Real-Time Quantitative PCR

Neuroretinas of all experimental conditions were submerged in RNA stabilizing solution (RNAlater, Invitrogen, CA, United States) and stored at −80°C until processing. RNA extraction was performed using Trizol reagent (Invitrogen, CA, United States) following the manufacturer’s instructions. The purity and quantity of the RNA were determined using a spectrophotometer (NanoDrop 2000, Thermo Fisher, MA, United States).

According to the manufacturer manual, the synthesis of the complementary DNA (cDNA) was performed through reverse transcription of the extracted mRNA using the High-Capacity cDNA Reverse Transcription Kit (Applied Biosystems, CA, United States). The relative quantitative real-time polymerase chain reaction (qPCR) was performed using the SYBR Green PCR master mix (Applied Biosystems, CA, United States) in the Applied Biosystems 7500 Real-Time PCR System (Applied Biosystems, CA, United States). Specific porcine primers were used to analyze the relative mRNA expression for apoptosis-related genes (*Pseudokinase mixed lineage kinase domain-like*, *MLKL; receptor-interacting serine/threonine-protein kinase 1 and 3*, *RIPK1 and RIPK3*) necroptosis-related genes (*BCL2 associated X*, *BAX; B-cell lymphoma 2*, *BCL2; cleaved caspase 3*, *8 and 9*, *CASP3*, *CASP8*, *and CASP9*) and autophagy-related genes (*mammalian Target of Rapamycin*, *mTOR; moesin-like BCL2 interacting protein*, *BCLIN1; microtubule-associated protein 1A/1B-light chain 3*, *LC3B; autophagy-related 7*, *ATG7; sequestosome1*, *SQSTM1*) (Table 1). The following cycle conditions were applied: 95°C for 10 mins, 40 cycles of 95°C for 15 s, 60°C for 1 min, and including a final melting curve step. *GAPDH* was used as a housekeeping gene to normalize the expression level of mRNA. Finally, we determined the threshold cycle for each reaction, and gene expression was quantified using the 2^–ΔΔCt^ method (Livak and Schmittgen, 2001). We performed all the qPCR assays in triplicate for each experimental condition.

**TABLE 1:** Gene-specific primer sequences for real-time quantitative PCR.

Gene	Forward primer (5′–3′)	Reverse primer (5′–3′)
*ATG7*	CGGGAACACTGTATAACACC	TCCTGCAAACGTCAAGAGGA
*BAX*	GAAGTTGAGCGAGTGTCT	AGTTGAAGTTGCCGTCAG
*BCL2*	TCATGAGTGTCAACTACCTG	TTGAGGAAGAGTAGGCTGTG
*BECLIN1*	AGGAGCTGCCGTTGTACTGTTCT	TGCTGCACACAGTCCAGGAA
*CASP3*	GGATTGAGACGGACAGTG	TTCGCCAGGAATAGTAACC
*CASP8*	GCATCATCTACGGCTCTG	ATAGGCTTCTGTCTGTTCTG
*CASP9*	ACAGATGGATGTCCTGTGTC	ACCCATGGTCTTTCTGTTCC
*LC3B*	AACGAAATTCCTGGTGCCTGA	AAGGCTTGGTTAGCATTGAGCTG
*MLKL*	CCCAGTTGCAGGAGGTCATT	CACGTGGCTTCACAAAAGGG
*mTOR*	ACTGGTTTCCAGCTCAGATG	TGGCAAATCTTCCGATTCGG
*RIPK1*	GGAGCATAACGAGCAGCGGAAG	CCAGAGCACGATGGCGAAGC
*RIPK3*	CATCGGGACCTCAAACCCTC	TCCCTGAAATGTGGACAGGC
*SOD1*	CAGTGTTAGTAACGGGAACC	ACACCATCTTTGCCAGCAGT
*SQSTM1*	CGCTTCAGCTTCTGTTTCAG	TACGACATCGCCATCGTCAGTT

### Immunohistochemical Characterization of Reactive Gliosis, Programmed Cell Death, and Autophagy

Neuroretinal explants were fixed with 4% paraformaldehyde (Panreac Quimica, Barcelona, Spain) for 24 h at 4°C. Then, they were subjected to a sucrose concentration gradient starting from 15, 20, and 30% of sucrose diluted in phosphatase-buffered saline (PBS) for 2 h at 4°C each step. Finally, explants were embedded in the Tissue-Tek OCT compound (Sakura Finetek Europe, Alphen, Netherlands) and cut into 5 μm cryosections using a cryostat (CM1900, Leica Microsystems Wetzlar, Germany).

Cryosections were washed in PBS and blocked [4% goat serum (Sigma-Aldrich) and 0.3% of Triton X-100 (Sigma-Aldrich) in PBS] for 2 h at room temperature (RT). Then, primary antibodies were incubated as described in Table 2. Molecular markers related to reactive gliosis [Glial fibrillary acidic protein (GFAP)], apoptosis [cleaved caspase-3, (Casp3)], necroptosis [Mixed lineage kinase domain-like protein (MLKL), Receptor-interacting serine/threonine-protein kinase 1 (RIPK1)] and autophagy [Nucleoporine P62 (p62), Microtubule-associated protein 1A/1B-light chain 3 (LC3B)] were evaluated. Afterward, species-specific secondary antibodies anti-rabbit IgG Alexa Fluor™ 568 goat (Thermo Fisher, MA, United States) and anti-mouse IgG Alexa Fluor™ 488 goat (Thermo Fisher, MA, United States) were incubated for 2 h at RT. Then, immunostaining with DAPI (4′,6-diamidino-2-fenilindol) was performed to visualize the nuclei. Subsequently, the samples were mounted using a fluorescent mounting medium (Dako, Glostrup, Denmark) and a coverslipped. All the immunohistochemical analyzes were performed in triplicate for each experimental condition, and control slides in which primary antibodies were omitted were processed in parallel.

**TABLE 2:** Primary and secondary antibodies and their experimental conditions.

Molecular marker	Primary antibody	Working dilution	Incubation time	Incubation temperature	Secondary antibody (1:200, 1 h, RT)
Glial fibrillary acidic protein (GFAP)	Polyclonal rabbit DakoCytomation Inc., n1506 (Glostrup, Denmark)	1:250	1 h	RT	Anti-rabbit IgG Alexa Fluor™ 568 goat (Thermo Fisher, MA, United States)
Cleaved caspase-3 (Casp3)	Polyclonal rabbit Cell Signaling Technology, D175 (MA, United States)	1:100	24 h	4°C	Anti-rabbit IgG Alexa Fluor™ 568 goat (Thermo Fisher, MA, United States)
TdT-mediated dUTP nick-end labeling (TUNEL) detection kit	- Roche Diagnostics, 11684795910 (Mannheim, Germany)	1:10	1 h	RT	–
Receptor-interacting serine/threonine-protein kinase 1 (Ripk1)	Polyclonal rabbit Sigma-Aldrich, SAB3500420 (Saint Louis, MO, United States)	1:200	24 h	4°C	Anti-rabbit IgG Alexa Fluor™ 568 goat (Thermo Fisher, MA, United States)
Mixed lineage kinase domain-like protein (MLKL)	Polyclonal mouse Sigma-Aldrich, SAB1408428 (Saint Louis, MO, United States)	1:200	24 h	4°C	Anti-mouse IgG Alexa Fluor™ 488 goat (Thermo Fisher, MA, United States)
Nucleoporin P62 (p62)	Polyclonal mouse Abcam, AB56416 (Cambridge, United Kingdom)	1:150	24 h	4°C	Anti-mouse IgG Alexa Fluor™ 488 goat (Thermo Fisher, MA, United States)
Microtubule-associated protein 1A/1B-light chain 3 (LC3B)	Polyclonal rabbit Novus Biological LL, NB600-1384 (CO, United States)	1:200	24 h	4°C	Anti-rabbit IgG Alexa Fluor™ 568 goat (Thermo Fisher, MA, United States)

*RT, room temperature.*

### TdT-Mediated dUTP Nick-End Labeling Immunohistochemical Analysis and Quantification

The TdT-mediated dUTP nick-end labeling (TUNEL) kit for detecting apoptosis at the single-cell level is based on labeling the DNA strand breaks. It was performed using a direct immunofluorescence protocol described in the manufacturer instructions. Cryosections were washed in PBS and blocked [4% goat serum (Sigma-Aldrich), 0.3% of Triton X-100 (Sigma-Aldrich) in PBS] for 2 h at RT. Then, TUNEL was incubated under the conditions detailed in Table 2. Afterward, DAPI immunostaining was used to visualize nuclei. We performed TUNEL analysis in triplicate for each experimental condition and control slides in which primary antibodies were omitted processed in parallel.

To quantify TUNEL immunoexpression, immunofluorescence micrographs (40× images; *n* = 12 sections per sample) were acquired at the same exposure, intensity, and gain levels. Afterward, each nuclear layer manually counted the TUNEL-stained nuclei using the software ImageJ (1.49 version, National Institutes of Health, Bethesda, MD, United States). Finally, the TUNEL labeled nuclei were correlated with the total DAPI-stained nuclei to obtain quantifiable results expressed as arbitrary units (AU).

### Immunofluorescence Micrographs Imaging

Immunofluorescence micrographs were captured using a LEICA TCS SP8 LIGHTNING confocal microscope (Leica Microsystems, Wetzlar, Germany) and analyzed with LEICA LAS AF software. Finally, processing and designing of figures were performed using the Pixelmator 3.8.2 Phoenix (Pixelmator Team, Vilnius, Lithuania).

### Statistical Analysis

Descriptive statistical analysis presented continuous variables in terms of mean and standard deviation (SD). Sample normality was evaluated using the Kolmogorov-Smirnov test. The parametric variables were analyzed using a *t*-test in the case of two groups and analysis of variance (ANOVA) for more than two. Meanwhile, non-parametric variables were analyzed using the Mann-Whitney *U* test for two groups and the Kruskal-Wallis test in the case of more than two groups. A confidence interval of 95% (*p* < 0.05) was considered to conclude significant differences between variables. Finally, the statistical analysis was performed using the SPSS version 22.0 statistical package (SPSS, Chicago, IL, United States).

## Results

### Reactive Gliosis

**FIGURE 1:**
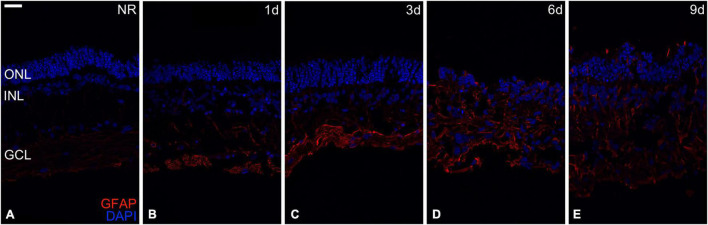
Reactive gliosis evaluation. Reactive gliosis analysis in the Glial fibrillary acidic protein (GFAP) immunoexpression during days 0, 1, 3, 6, and 9 of neuroretinal degeneration **(A–E)**. GFAP immunoexpression for Fresh neuroretina **(A)**, day 1 **(B)**, day 3 **(C)**, day 6 **(D)**, and day 9 of neuroretinal degeneration **(E)**. Scale bar: 25 μm.

As a sign of retinal neurodegeneration (Cuenca et al., 2014), reactive gliosis was assessed using GFAP immunoexpression, upregulated in Müller cells during retinal degeneration (Barber et al., 1998). GFAP immunoexpression in fresh neuroretina was mainly restricted to the inner neuroretina; then, increasing cultivation time reached the outer retina, being detectable in all neuroretinal layers from day 6. Besides, nuclei organization and distribution became more heterogeneous from days 1 to 9, retinal layers were hard to differentiate since day 6 (Figure 1).

### Apoptosis Analysis

The relative mRNA expression of the apoptosis-related genes *BAX*, *BCL2*, *CASP3*, *CASP8*, and *CASP9* was analyzed to study the events associated with the apoptosis process during spontaneous retinal degeneration. In addition, the immunoexpression of the Casp3 protein and quantification of the TUNEL apoptosis detection kit was performed.

**FIGURE 2:**
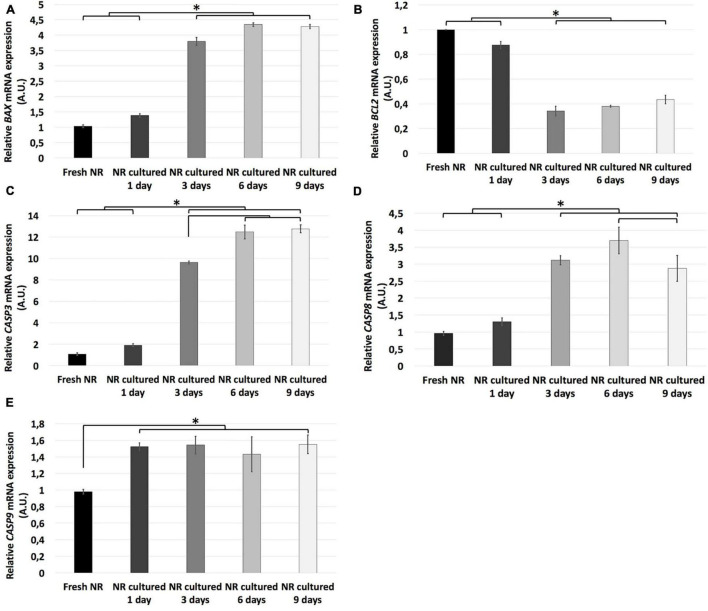
Relative mRNA expression of apoptosis-related genes during neuroretinal degeneration. Relative mRNA expression of the apoptosis-related genes: *BAX* **(A)**, *BCL2* **(B)**, *CASP3* **(C)**, *CASP8* **(D)**, and *CASP9* **(E)** during days 0, 1, 3, 6, and 9 of neuroretinal degeneration. *p < 0.05, AU, arbitrary units.

Relative mRNA expression of the *BAX*, *CASP3*, *CASP8*, and *CASP9* genes was significantly higher in all the experimental conditions than fresh neuroretina. *BAX*, *CASP3*, *and CASP9* mRNA relative expression remained at the same level from fresh to day 1 and increased significantly from day 1 to 3. From day 3 to 6, the relative RNA expression of *CASP3* increased significantly, and *CASP8* maintained the same expression. From day 6 to 9, *CASP 8* decreased significantly, and *CASP3* remained the same. In the case of *CASP9* mRNA relative expression, there were no significant differences after day 1. Besides, the relative mRNA expression of *BCL2* was significantly increased in fresh and day 1 compared to all the other experimental conditions; then, it remained the same after day 3 (Figure 2).

**FIGURE 3:**
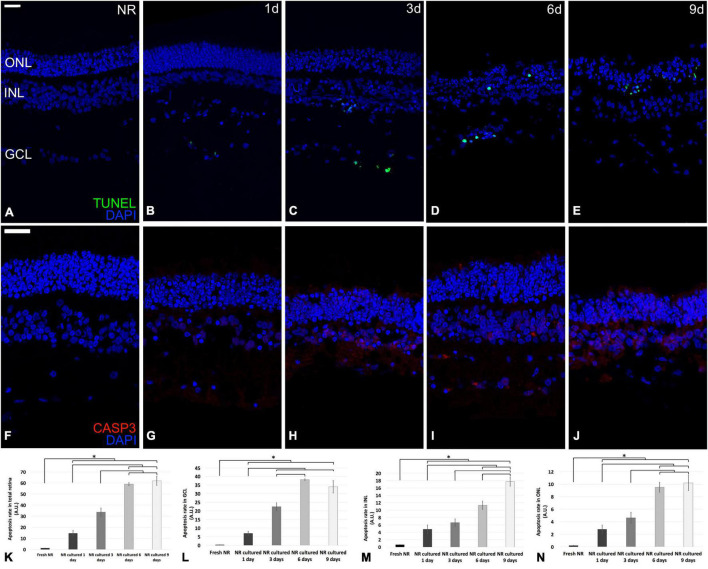
Immunohistochemistry analysis and quantification of apoptosis-related molecular markers during neuroretinal degeneration. Immunoexpression of the apoptosis-related protein cleaved caspase-3 and the molecular marker TUNEL during days 0, 1, 3, 6, and 9 of neuroretinal degeneration and quantification of the TUNEL labeled nuclei for each retinal nuclear layer. TUNEL immunoexpression for Fresh neuroretina **(A)**, day 1 **(B)**, day 3 **(C)**, day 6 **(D)**, and day 9 of neuroretinal degeneration **(E)**. Casp3 immunoexpression for Fresh neuroretina **(F)**, day 1 **(G)**, day 3 **(H)**, day 6 **(I)**, and day 9 of neuroretinal degeneration **(J)**. Quantification of TUNEL labeled nuclei for total retina **(K)**, GCL **(L)**, INL **(M)**, and ONL **(N)**. *p < 0.05, AU, arbitrary units; GCL, ganglion cell layer; INL, inner nuclear layer; ONL, outer nuclear layer. Scale bar: 25 μm.

Casp3 is the primary execution apoptotic protein common for all the apoptosis pathways (Cuenca et al., 2014); meanwhile, the TUNEL detection kit labels DNA strand breaks in cells that undergo apoptosis. Both were mostly restricted to the GCL on day 1, reached the INL on day 3, and the ONL on days 6 and 9. However, TUNEL seemed to be mostly restricted to ONL at day 9; meanwhile, Casp 3 were distributed through the whole neuroretina (Figures 3A–J). The TUNEL quantification showed that the apoptosis rate in the total retina and all nuclear retinal layers, in an independent way (GCL, INL, and ONL), increased progressively from fresh to day 6. From day 6 to 9, the apoptosis rate in the total retina, GCL, and ONL remained the same and raised in the case of INL. The apoptosis peak in the whole retina, GCL, and ONL were at day 6; meanwhile, in the INL, the apoptosis rate peak was at day 9 (Figures 3K–N).

### Necroptosis Analysis

We assessed the necroptotic events by analyzing the relative mRNA expression of the *MLKL*, *RIPK1*, *and RIPK3* genes and the immunoexpression of the proteins MLKL and RIPK1.

**FIGURE 4:**
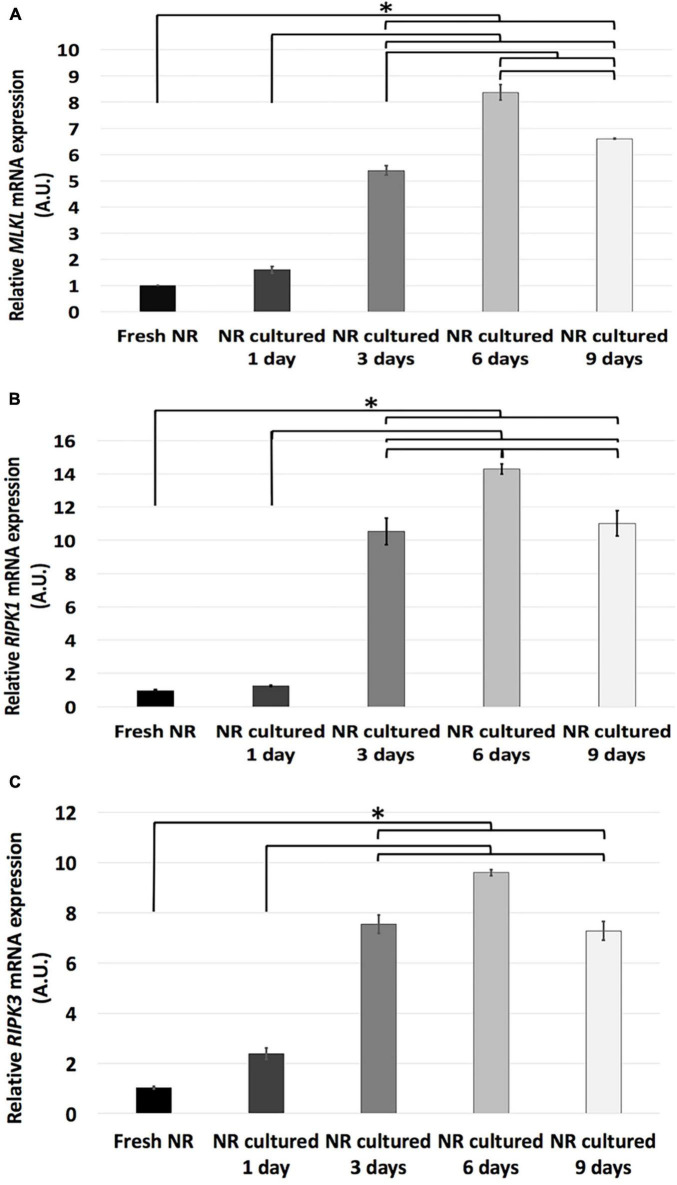
Relative mRNA expression of necroptosis-related genes during neuroretinal degeneration. Relative mRNA expression of the necroptosis-related genes: *MLKL* **(A)**, *RIPK1* **(B)**, *RIPK3* **(C)** during days 0, 1, 3, 6, and 9 of neuroretinal degeneration. *p < 0.05, AU, arbitrary units.

The results showed that relative mRNA expression of necroptosis-related genes studied (*MLKL*, *RIPK1*, *and RIPK3*) remained the same from fresh to day 1 and significantly increased from day 1 to 3. In the case of *MLKL and RIPK1* genes, its relative mRNA expression significantly increased from day 3 to 6, and both decreased from day 6 to 9. Meanwhile, the relative mRNA expression of *RIPK3* remained the same since days 3 to 9 (Figure 4).

**FIGURE 5:**
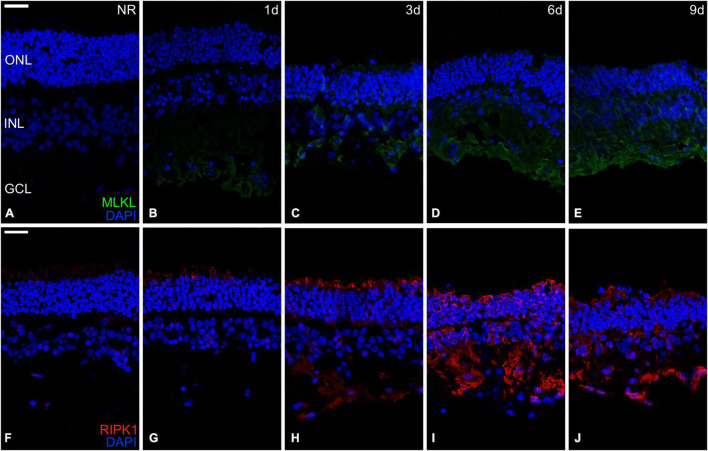
Immunohistochemistry analysis of necroptosis-related proteins during neuroretinal degeneration. Immunoexpression of the apoptosis-related proteins Mixed lineage kinase domain-like protein (MLKL) and Receptor-interacting serine/threonine-protein kinase 1 (RIPK1) during days 0, 1, 3, 6, and 9 of neuroretinal degeneration. MLKL immunoexpression for Fresh neuroretina **(A)**, day 1 **(B)**, day 3 **(C)**, day 6 **(D)**, and day 9 of neuroretinal degeneration **(E)**. RIPK1 immunoexpression for Fresh neuroretina **(F)**, day 1 **(G)**, day 3 **(H)**, day 6 **(I)**, and day 9 of neuroretinal degeneration **(J)**. Scale bar: 25 μm.

The protein RIPK1 induces phosphorylation of the MLKL, which produces the formation of cell membrane pores leading to cell death (Vandenabeele et al., 2010). The immunoexpression of both proteins was not detectable in fresh neuroretinas. The immunoexpression distribution of the MLKL protein begins at the CGL and then proliferates to the most external parts, reaching the whole neuroretina at day. Meanwhile, the immunoexpression of the RIPK1 protein started its proliferation from the photoreceptors’ outer segments heading toward the most internal parts, reaching the whole neuroretina at day 3 (Figure 5).

### Autophagy Analysis

Regarding autophagy, the relative mRNA expression of *ATG7*, *BCLIN1*, *LC3B*, *mTOR*, *and SQSTM1* genes and the immunoexpression of the LC3B and p62 proteins were analyzed.

**FIGURE 6:**
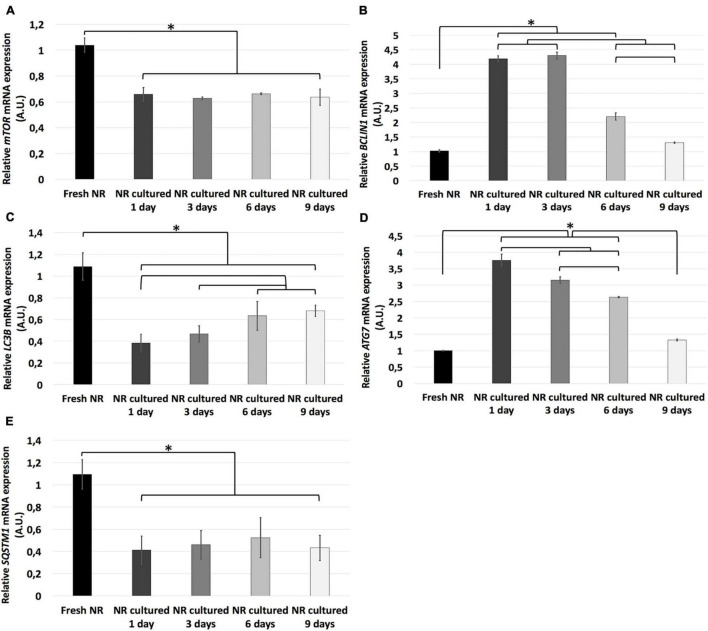
Relative mRNA expression of autophagy-related genes during neuroretinal degeneration. Relative mRNA expression of the autophagy-related genes: *LC3B* **(A)**, *BCLIN1* **(B)**, *LC3B* **(C)**, *ATG7* **(D)**, and *SQSTM1* **(E)** during days 0, 1, 3, 6, and 9 of neuroretinal degeneration. *p < 0.05, AU, arbitrary units.

Relative mRNA expression of *mTOR*, *LC3B*, *and SQSTM1* genes was significantly higher in fresh neuroretinas than in any other experimental conditions, where there were no significant differences between *mTOR and SQSTM1* genes. In the case of *LC3B* mRNA relative expression, it increased progressively from day 1 to 6 and remained stable from day 6 to 9. In contrast, the relative mRNA expression of *ATG7 and BCLIN1* genes significantly increase from fresh to day 1, where it reaches its expression peak. The mRNA relative expression of *BCLIN1* remained the same from day 1 to 3 and progressively decreased from day 3 to 9. Finally, *ATG7* reduced gradually from day 1 to 9 (Figure 6).

**FIGURE 7:**
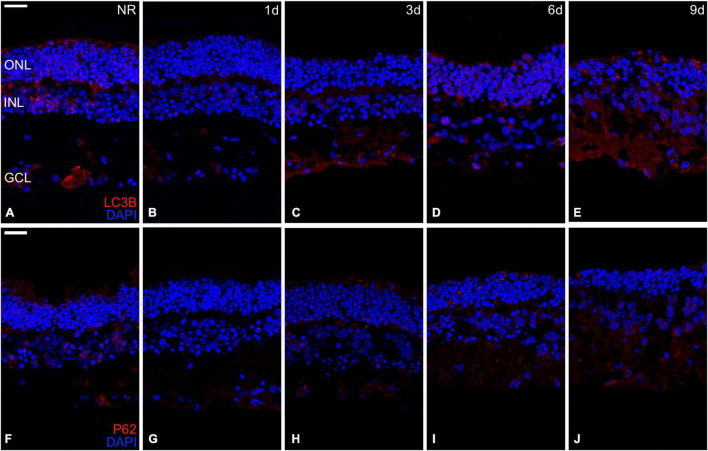
Immunohistochemistry analysis of autophagy-related proteins during neuroretinal degeneration. Immunoexpression of the autophagy-related proteins Microtubule-associated protein 1A/1B-light chain 3 (LC3B) and Nucleoporine P62 (p62) during days 0, 1, 3, 6, and 9 of neuroretinal degeneration. LC3B immunoexpression for Fresh neuroretina **(A)**, day 1 **(B)**, day 3 **(C)**, day 6 **(D)**, and day 9 of neuroretinal degeneration **(E)**. P62 immunoexpression for Fresh neuroretina **(F)**, day 1 **(G)**, day 3 **(H)**, day 6 **(I)**, and day 9 of neuroretinal degeneration **(J)**. Scale bar: 25 μm.

The proteins LC3B and p62 are essential for autophagosome formation, which delivers cell components to lysosomal degradation (Levine and Kroemer, 2008). The immunoexpression of both proteins was present in fresh days 3, 6, and 9, being almost undetectable at day 1 (Figure 7).

## Discussion

As mentioned, RND is the leading cause of irreversible blindness worldwide, and current therapeutic options are only palliative or too specific and expensive. But this group of diseases, independently of its etiology, have closely related pathophysiology at the cellular and molecular level (Cuenca et al., 2014). In that sense, unspecific neuroprotective therapies to stop or slow down their progressions, such as targeting pathophysiological molecular mechanisms related to cell death and autophagy, seem to be an attractive therapeutic alternative. Hence, it is necessary to characterize as profoundly as possible the molecular pathways that converge during retinal degeneration to target the most relevant molecules during the development of therapeutic options. For the first time, our study describes and provides a comprehensive evaluation of the central programmed cell death and autophagy mechanisms during *in vitro* neuroretinal degeneration. The *in vitro* study presented here is the first step for future *in vivo* studies that focuses on the molecular pathways analyzed here for subsequent translation to clinical practice.

However, this study incurs certain limitations. Firstly, our *in vitro* retinal neurodegeneration model lacks an RPE layer, vascularization, and the RGC were axotomized. Secondly, spontaneous neuroretinal degeneration does not represent a retinal disease from a clinical point of view. However, even with these limitations, this work contributes significantly to understanding the crucial role of programmed cell death and autophagy during retinal neurodegeneration.

The retinal spontaneous degeneration model used for this study is an excellent alternative to study the molecular and cellular changes that occur during retinal degenerative diseases since they retain the complex neuroretinal architecture, interactions of neuroretinal cells, and mimic the *in situ* responses under controlled conditions (Li et al., 2018; Murali et al., 2019; Fernandez-Bueno and Usategui-Martin, 2021). However, it presents the drawback of lacking both retinal and choroidal vascularization, the axotomy of retinal ganglion cells, and the absence of retinal pigmented epithelium, for that reason, this can only resemble the degeneration of the neuroretina, a joint event in most retinal neurodegenerative diseases.

One of the most representative structural changes during retinal degeneration is reactive gliosis, which means the proliferation of glial cells to neuroprotect retinal neurons, which can be detrimental if the cause is not eliminated (Cuenca et al., 2014). The upregulation of GFAP in Müller cells has been previously described in animal models of diabetic retinopathy (Barber et al., 1998), RP (Fernandez-Sanchez et al., 2010), and glaucoma (Fernandez-Sanchez et al., 2013), and *in vitro* by our research group (Fernandez-Bueno et al., 2008, 2012, 2013; Di Lauro et al., 2016; Labrador-Velandia et al., 2019; Usategui-Martín et al., 2020) by using the same organotypic neuroretina model, our results presented here to add more evidence to this premise.

The primary programmed cell death mechanism during retinal neurodegeneration is apoptosis. However, recent studies have proved and highlighted the interrelationship between apoptosis, other cell death mechanisms, and autophagy (Newton and Megaw, 2020). Apoptosis is a programmed cell death mechanism that removes potentially dangerous cells without damaging the surrounding tissues. It can be mediated through the caspase-dependent or -independent pathways (Doonan et al., 2005; Kroemer and Martin, 2005; Murakami et al., 2013). This study evaluated apoptosis-related genes implicated in the caspase-dependent pathway. They play primary roles in the extrinsic (*Casp8*), intrinsic (*BAX*, *BCL2*, and *Casp9*), and execution (*Casp3*) pathways (Cuenca et al., 2014). Additionally, the balance between pro-apoptotic (*B*ax) and anti-apoptotic (*BCL2*) genes was evaluated (Singh et al., 2019). Our results showed that until day 1 of retinal spontaneous *in vitro* neurodegeneration, the relative mRNA expression of the anti-apoptotic *BCL2* gene was predominant; however, since day 3, the anti-apoptotic mechanisms are inhibited both extrinsic and intrinsic caspase-dependent pathways are activated. Then, apoptosis-related genes increased progressively until day 9, even when most of the retinal architecture had been lost. At the protein level, we corroborated this analysis with the immunoexpression curve of TUNEL and Casp3, which progressed simultaneously to the activation of the apoptosis-related genes. Besides, TUNEL quantification showed that apoptosis seems to be proportionally activated in all retinal nuclear layers.

Apoptosis and necrosis are two primary cell death mechanisms (Kroemer et al., 2009). Necrosis has been considered an unregulated form of cell death; however, recent evidence suggests that it can be induced by regulated signal transduction pathways (Festjens et al., 2006; Golstein and Kroemer, 2007). Regulated necrosis includes several cell-death modalities, although necroptosis is the best characterized. Necroptosis is a programmed cell death mechanism that implies disruption of the cell membrane, causing liberation of damage-associated molecular patterns that induce inflammation (Pasparakis and Vandenabeele, 2015). Necroptosis is mainly regulated by the three genes evaluated in this study (*RIPK1*, *RIPK3*, *and MLKL*). The interaction between RIPK1 and RIPK3 activates the phosphorylation and oligomerization of the MLKL that induce the formation of membrane pores, resulting in the loss of membrane integrity, causing cell death (Degterev et al., 2005; Vandenabeele et al., 2010; Dondelinger et al., 2014). Our results showed that necroptosis-related proteins immunoexpression could be detected since day 1; however, there has been a substantial increase in relative mRNA expression and protein distribution of necroptosis since day 3.

The pro-apoptotic and pro-necroptotic proteins’ upregulation during neuroretinal degeneration is correlated with the alteration in nuclei organization and distribution observed from days 1 to 9. Besides, a reduced number of nuclei and an increment of TUNEL labeled nuclei during neuroretinal degeneration is apparently, suggesting that the up-regulation of these proteins could be associated with retinal cells loss; however, to confirm this, it would be more accurate to count manually the number of nuclei per μm^2^ in every nuclear layer individually on hematoxylin-eosin stained neuroretinal non-serial sections, as in our previous work (Usategui-Martín et al., 2020).

The interrelationship between necroptosis and apoptosis has been described *in vivo* in a retinal detachment model. It exhibited overexpression of necroptosis as a compensatory mechanism when apoptosis is inhibited. Besides, it has been demonstrated that necroptosis is an effective cell death mechanism involving rod and cone degeneration during RP pathogenesis using *in vivo* animal models (Murakami et al., 2012; Sato et al., 2013; Yang et al., 2018; Viringipurampeer et al., 2019). Our results showed that the activation of necroptosis occurs parallel to apoptosis *in vitro* after the anti-apoptotic mechanisms were suppressed, having a substantial increase by day 3 and continue growing progressively until day 9. This simultaneous activation of apoptosis and necroptosis *in vitro* reinforces the hypothesized interrelationship between them during retinal neurodegeneration.

Autophagy is a lysosome-mediated pathway that degenerates and recycles cells’ defective proteins and organelles. It is essential for survival, differentiation, development, and homeostasis (Levine and Kroemer, 2008; Newton and Megaw, 2020). For that reason, it occurs at low basal levels in virtually any cell; however, it could be rapidly upregulated when the cell needs to generate intracellular energy to undergo structural remodeling, induced by oxidative stress or protein aggregate accumulation, events that are crucial in retinal degeneration (Klionsky, 2007; Maiuri et al., 2007; Mizushima and Klionsky, 2007; Rubinsztein et al., 2007; Cuenca et al., 2014). Autophagy requires the formation of a double-membrane vesicle where the target is encapsulated, called the autophagosome, and its subsequent fusion with the lysosomes degrades the target (Levine and Kroemer, 2008). The genes selected for this study is associated with crucial phases of autophagy, including the isolation of the future membrane of the autophagosome (*BCLN1*), elongation and formation of the membrane (*LC3B and SQSTM1*), and *ATG7* by the activation of *LC3B*; besides, we evaluated the primary inhibitory signal that shuts off autophagy in the presence of growth factors or nutrients (*mTOR*) (Klionsky, 2007; Mizushima and Klionsky, 2007; Liu et al., 2013). Our results revealed how autophagy processes are modulated during *in vitro* neuroretinal degeneration, supporting the hypothesis that autophagy may play an essential role in the RND pathophysiology.

P62/SQTM1 plays a fundamental role in activating and organizing autophagy (Wang et al., 2014). Furthermore, P62 and LC3B, both components of the autophagosome membrane, are decreased through autophagy-mediated degradation during the process (Viiri et al., 2013). Our results are highly correlated with this premise. Both P62 and LC3B proteins can be detected through immunostaining in fresh neuroretinas, but not at day 1, then both recover their immunoreactivity since day 3. Besides, the anti-autophagic signal mediated by the *mTOR* gene is higher in fresh neuroretinas and decreased since day 1. These results suggest that, unlike apoptosis and necroptosis, the inhibition of anti-autophagy factors may start since day 1 of spontaneous *in vitro* neuroretinal degeneration. That autophagy could reach its maximum expression by day 1, before apoptosis and necroptosis (by day 3). These findings correlate with the previous idea that autophagy leads to apoptosis when severe cellular injury (Mukhopadhyay et al., 2001). Besides, the role of autophagy during *in vitro* RND has been tested *in vivo*, specifically in models of RP (Rodríguez-Muela et al., 2015; Yao et al., 2018; Li et al., 2019; Qiu et al., 2019) and AMD (Mitter et al., 2012; Kaarniranta et al., 2017). In that sense, autophagy plays a leading role in the early stages of *in vitro* neuroretinal degeneration.

The mechanism described in this study may cause changes in retinal cells’ morphology and connectivity, leading to retinal remodeling, which is a joint event in many RND (Cuenca et al., 2014). The up-regulation of apoptosis has been associated with atrophic and neovascular forms of AMD (Ramírez et al., 2001; Dunaief et al., 2002; Bhutto and Lutty, 2012), RP (Comitato et al., 2014), and diabetic retinopathy (Martin et al., 2004). Besides, over-expression of necroptosis has been implicated in models of RP (Murakami et al., 2012; Sato et al., 2013; Yang et al., 2017; Viringipurampeer et al., 2019), retinal detachment (Dong and Sun, 2011), retinal ischemia-reperfusion injury (Rosenbaum et al., 2010), and achromatopsia (Viringipurampeer et al., 2014). On the other hand, the curve modulation of autophagy described by our results may be consistent with the association of the down-regulation of the mTOR pathway and retinal cell death through secondary activation of apoptosis, suggested in RP models (Newton and Megaw, 2020).

Our results support that non-apoptotic cell death mechanisms are essential during neuroretinal degeneration. Besides, the time course modulation of the three mechanisms described could help target a specific cell death mechanism depending on the disease progression. Moreover, this study provides new information about the modulation of autophagy during retinal neurodegeneration; it has been demonstrated that autophagy has significant implications during retinal at the therapeutic level using drugs such as rapamycin, an autophagy agonist (Rubinsztein et al., 2007) or Rasagiline, anti-apoptotic and autophagy modulating medication (Eigeldinger-Berthou et al., 2012).

In conclusion, this study provides new information about molecular signaling of cell death mechanisms along the neuroretinal degeneration process and reinforces the idea that cell death mechanisms could be interconnected. This information is relevant to the future development of an integral neuroprotective therapy that thoroughly covers the cell death process during neurodegenerative retinal diseases. Finally, we consider that effective neuroprotective treatment must simultaneously prevent the activation of main cell death mechanisms to be effective, as observed in this study.

## Data Availability Statement

The raw data supporting the conclusions of this article will be made available by the authors, without undue reservation.

## Author Contributions

RU-M and IF-B designed the study. IF-B coordinated the study. RU-M, KP-N, NG-C, LH-R, FG-P, and IF-B generated research data. KP-N, RU-M, JP JR-C, RG-S, and IF-B analyzed and discussed the results. KP-N and IF-B wrote the manuscript. All authors reviewed the manuscript, contributed to discussion and approved the final version of the manuscript.

## Conflict of Interest

The authors declare that the research was conducted in the absence of any commercial or financial relationships that could be construed as a potential conflict of interest.

## Publisher’s Note

All claims expressed in this article are solely those of the authors and do not necessarily represent those of their affiliated organizations, or those of the publisher, the editors and the reviewers. Any product that may be evaluated in this article, or claim that may be made by its manufacturer, is not guaranteed or endorsed by the publisher.

## Supplementary Material

Supplementary Figure 1 Dissected areas from pig retinas. *Area centralis* (AC) and optic nerve (OP) in **(A)** dissected posterior segment in the porcine globe; **(B)** dissected porcine neuroretina in Petri dish; and **(C)** neuroretina schematic drawing. AC: *area centralis*; ON: optic nerve.
