# Supplementary figures and images for: Programmed Cell Death and Autophagy in an in vitro Model of Spontaneous Neuroretinal Degeneration
Source: Front Neuroanat. 2022 Feb 11;16:812487. doi: 10.3389/fnana.2022.812487 (PMC8873173; doi:10.3389/fnana.2022.812487)

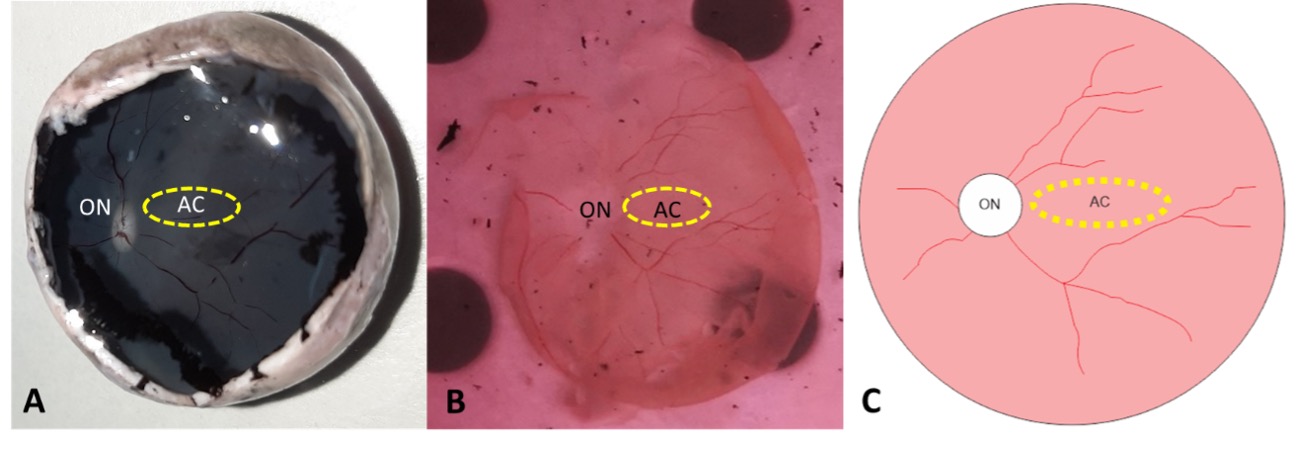

Supplement: Supplementary Figure 1 — Dissected areas from pig retinas. Area centralis (AC) and optic nerve (OP) in (A) dissected posterior segment in the porcine globe; (B) dissected porcine neuroretina in Petri dish; and (C) neuroretina schematic drawing. AC: area centralis; ON: optic nerve. [file Image_1.JPEG]
